# Pharmacodynamic, pharmacokinetic, and phase 1a study of bisthianostat, a novel histone deacetylase inhibitor, for the treatment of relapsed or refractory multiple myeloma

**DOI:** 10.1038/s41401-021-00728-y

**Published:** 2021-08-02

**Authors:** Yu-bo Zhou, Yang-ming Zhang, Hong-hui Huang, Li-jing Shen, Xiao-feng Han, Xiao-bei Hu, Song-da Yu, An-hui Gao, Li Sheng, Ming-bo Su, Xiao-li Wei, Yue Zhang, Yi-fan Zhang, Zhi-wei Gao, Xiao-yan Chen, Fa-jun Nan, Jia Li, Jian Hou

**Affiliations:** 1 National Center for New Drug Screening, State Key Laboratory of Drug Research, Shanghai Institute of Materia Medica, Chinese Academy of Sciences, Shanghai, 201203 China; 2 University of Chinese Academy of Sciences, Beijing, 100049 China; 3 Yantai Key Laboratory of Nanomedicine & Advanced Preparations, Yantai Institute of Materia Medica, Yantai, 264000 China; 4 Department of Hematology, Renji Hospital, Shanghai Jiaotong University School of Medicine, Shanghai, 200127 China; 5 Shanghai Center for Drug Metabolism and Pharmacokinetics Research, Shanghai, 201203 China

**Keywords:** multiple myeloma, HDAC inhibitor, bisthianostat, pharmacodynamics, pharmacokinetics, phase 1a clinical trial, antitumor drug

## Abstract

HDAC inhibitors (HDACis) have been intensively studied for their roles and potential as drug targets in T-cell lymphomas and other hematologic malignancies. Bisthianostat is a novel bisthiazole-based pan-HDACi evolved from natural HDACi largazole. Here, we report the preclinical study of bisthianostat alone and in combination with bortezomib in the treatment of multiple myeloma (MM), as well as preliminary first-in-human findings from an ongoing phase 1a study. Bisthianostat dose dependently induced acetylation of tubulin and H3 and increased PARP cleavage and apoptosis in RPMI-8226 cells. In RPMI-8226 and MM.1S cell xenograft mouse models, oral administration of bisthianostat (50, 75, 100 mg·kg^-1^·d^-1^, bid) for 18 days dose dependently inhibited tumor growth. Furthermore, bisthianostat in combination with bortezomib displayed synergistic antitumor effect against RPMI-8226 and MM.1S cell in vitro and in vivo. Preclinical pharmacokinetic study showed bisthianostat was quickly absorbed with moderate oral bioavailability (*F*% = 16.9%–35.5%). Bisthianostat tended to distribute in blood with *V*_ss_ value of 0.31 L/kg. This distribution parameter might be beneficial to treat hematologic neoplasms such as MM with few side effects. In an ongoing phase 1a study, bisthianostat treatment was well tolerated and no grade 3/4 nonhematological adverse events (AEs) had occurred together with good pharmacokinetics profiles in eight patients with relapsed or refractory MM (R/R MM). The overall single-agent efficacy was modest, stable disease (SD) was identified in four (50%) patients at the end of first dosing cycle (day 28). These preliminary in-patient results suggest that bisthianostat is a promising HDACi drug with a comparable safety window in R/R MM, supporting for its further phase 1b clinical trial in combination with traditional MM therapies.

## Introduction

Histone deacetylases (HDACs) catalyze the removal of acetyl groups from the positively charged lysine residues in proteins [1], play a key role in the epigenetic regulation of gene expression, and are involved in many different types of cancer [2]. In recent years, HDACs have been fully investigated for their roles and potential as drug targets in T-cell lymphomas and other hematologic malignancies. Several small-molecule HDAC inhibitors (HDACis) have been approved, such as vorinostat (SAHA) for CTCL, romidepsin for CTCL and PTCL, belinostat for PTCL, and chidamide for PTCL [3–7], making HDACs as the most promising and successful target in epigenetic regulators [8].

Significant progress of HDACi in clinical indication is the approval of panobinostat (LBH-589) by US FDA as the first HDACi to treat relapsed or refractory multiple myeloma (R/R MM) in combination with bortezomib [9, 10]. MM is an incurable plasma cell malignancy as the second most common hematological malignancy. This disease remains incurable as nearly all patients will relapse and become refractory to established MM therapy. Thus, new treatment option for R/R MM is needed, particularly those with different mechanisms of action. One such approach is to inhibit HDAC and produce synergistic anti-myeloma activity via mechanisms of epigenetic modulations [11–13].

Bisthianostat (BIS, CF367-C) is a new type of bisthiazole-based pan-HDACi evolved from the thiazole–thiazoline cap group in natural HDACi largazole [14] and approved by CDE as a class 1.1 anticancers IND. BIS was developed via systematic structure–activity-relationship studies based on the in vitro activities, cellular activities, as well as in vivo efficacy, and then identified as a promising drug candidate for further clinical development.

Preclinical pharmacokinetic (PK) study showed that BIS was quickly absorbed with a moderate oral bioavailability (*F*% = 16.9%–35.5%). In R/R MM patients, BIS treatment was well tolerated and no grade 3/4 nonhematological AEs have occurred. It was rapidly absorbed after oral administration and eliminated quickly from plasma, with a *t*_max_ of 0.5–2.25 h and a *t*_1/2_ of about 4 h, respectively. The mean Cl/*F* was from 42.9 to 77.5 L/h, and *V*_d_/*F* was from 230 to 538 L/h. The overall efficacy was modest and stable disease (SD) was identified in four (50%) patients at the end of first dosing cycle (day 28). Notably, one patient in 200 mg cohort remained SD for up to nearly 7 months with single-agent BIS.

In this paper, we present the preclinical anti-MM activity of BIS, alone and in combination with bortezomib in vitro and in vivo, the preclinical PK, tissue distribution profiles, and preliminary first-in-human findings from CH-020PI study, an ongoing phase 1a study of BIS (trial registered at ClinicalTrial.gov: NCT03618602).

## Materials and methods

### Cell lines and reagents

Dexamethasone (Dex)-sensitive MM.1S and Dex-resistant MM.1R were obtained from the American Type Culture Collection (Manassas, VA, USA). RPMI-8226 and U266, NCI-H929, SKO, and LP1 human MM cells were provided by Changzheng Hospital, Shanghai. All MM cell lines were cultured with RPMI-1640 + 10% FBS medium. BIS was synthesized in Shanghai Institute of Materia Medica. BIS was dissolved first in DMSO at a concentration of 10 mM, and then in culture medium (0.5–8 μM) immediately before use. SAHA was purchased from MedExpress company.

Bortezomib was obtained from Selleck Chemicals for the in vitro studies. It was dissolved first in DMSO at a concentration of 20 mM, and then in culture medium before use. Bortezomib formulated with mannitol was dissolved in saline for the in vivo studies.

Histone H3, acetyl-histone H3 (Lys23), and poly(ADP-ribose) polymerase antibodies were obtained from Cell Signaling Technology (Beverly, MA, USA). Acetylated-α-tubulin (6-11B-1) and alpha-tubulin antibody were obtained from Santa Cruz Biotechnology (Santa Cruz, CA, USA).

### Cell viability and proliferation assays

The effect of BIS or SAHA on the viability of MM cell lines was assessed by measuring CCK8 (Dojindo Laboratories, Kumamoto, Japan) dye absorbance. MM cells ([0.5 − 1] × 10^4^ cells/well) were incubated in 96-well culture plates with medium and different concentrations of BIS or SAHA for 72 h at 37 °C and cell viability and proliferation were measured, as described in the CCK8 protocol.

### Cell apoptosis assay

RPMI-8226 cells (6 × 10^5^) were cultured in a standard (concentration free) medium or in a medium containing multiple concentrations of BIS for 48 h. Cells were harvested, washed, and stained with annexin V/propidium iodide (PI), according to the manufacturer’s product description. Annexin V^+^/PI^−^ apoptotic cells were enumerated using the BD Accuri™ C6 flow cytometer. The percentage of cells undergoing apoptosis was defined as the sum of early apoptosis (annexin V^+^) and late apoptosis (annexin V^+^ and PI^+^) cells.

### Western blotting (WB)

RPMI-8226 cells (6 × 10^5^) were cultured with different concentrations of BIS or SAHA for 24 h. Then, cells were washed with ice-cold PBS and lysed with the RIPA buffer for 30 min. Protein-matched lysates were boiled in Laemmli sample buffer. Equal amounts of protein were separated using SDS-PAGE and subsequently transferred onto a nitrocellulose membrane. The membranes were blocked with TBST buffer with 5% of nonfat dry milk and incubated in primary anti-human antibodies followed by secondary antibodies. The proteins of interest were visualized using the Li-Cor infrared imaging system (Odyssey).

### MM xenograft mouse model

To evaluate the in vivo anti-MM activity of BIS, female NOD/SCID mice were inoculated subcutaneously with 1 × 10^7^ MM.1S cells or RPMI-8226. Mice were purchased from Beijing Vital River Laboratory Animal Technology Co., Ltd. Animal use procedures were approved based on the guidelines established by the Committee for Laboratory Animal Research at the Shanghai Research Center for Model Organisms (IACUC approval number: 2013-0006). When tumors were measurable, mice were treated with *p.o*. BIS at different doses consecutively for 18 days; bortezomib 1 or 1.2 mg/kg biweekly (i.v) as positive control; or 0.5 mg/kg bortezomib in combination with different doses of BIS. Tumor size was measured every other day using calipers, and tumor volume was calculated with the formula: *V* = 0.5 (*a* × *b*^2^), where *a* is the long diameter of the tumor and *b* is the short diameter of the tumor. Mice were killed when the tumor reached 2 cm^3^ or was ulcerated. Tumor growth was evaluated from the first day of treatment until the end of experiment.

For PD analysis, mice were killed 1 h after treatment, tumor tissues were collected for WB analysis of acetylated α-tubulin and acetylated histone H3 levels, as before indicated.

### PK, tissue distribution studies in mice, and CYPs inhibition assessment

Preclinical PKs and distribution of BIS were assessed in ICR mice (25–30 g), which were purchased from Shanghai B&K Universal Group Limited (qualification no. SCXK (Hu) 2013-0016).

In PK study, 96 ICR mice were randomly divided into four groups, which received doses of 50, 100, and 200 mg/kg of BIS by *p.o.* route and 10 mg/kg by i.v. route via the tail vein, respectively. The sampling time points for both *p.o*. and i.v. doses were all set at pre dose and at 5 and 15 min, and 0.5, 1, 2, 4, 6, 8, 10, 12, 16, and 24 h post dose, and each time point was based on samples from six different mice (three male and three female). Blood samples (0.2 mL/sample) were collected into EDTA-K2 tubes, and then centrifuged for 5 min (11000 r/min). The separated plasma samples were stored at approximately −20 °C for further analysis using an HPLC/MS/MS method. The PK parameters were calculated based on the mean concentration profiles using a standard noncompartmental analysis (NCA) method for sparse sampling data, and the software for PK analysis was Phoenix WinNonlin 7.0 (Certara, Princeton, NJ, USA).

In tissue distribution study, 24 ICR mice received 100 mg/kg of BIS by *p.o.* route. They were euthanized at 0.5, 2, 8, and 24 h post dose (six animals at each time point) and tissues (such as brain, heart, liver, spleen, lungs, kidney, bladder, uterus and ovary, intestine, stomach, and pancreas) were collected. Tissue homogenate was made and stored at approximately −20 °C for further analysis using an HPLC/MS/MS method.

Human liver microsomes (BD Gentest, NJ, USA) were used to evaluate the inhibition potential of BIS on major cytochrome P450s by using specific CYP450 probe substrates. Human liver microsomes (0.5 mg/mL) were incubated at 37 °C with BIS at various concentrations (0.00–100 μM) with specific CYP450 probe substrates and NADPH (1 mM). LC-MS/MS method was used to determine the concentration of each specific metabolites and the value of IC_50_ was calculated to indicate the inhibition potential of BIS toward major CYPs.

### Clinical study design and objectives

CH-020PI was a first-in-human study based on a traditional 3 + 3 design. It was a phase 1a, single center, single-arm, open-label, dose-escalation study of single-agent BIS conducted in China. The first dose in human (100 mg) was calculated according to 1/5 of its preclinical NOAEL value in beagle dogs (NOAEL < 15 mg·kg^-1^·d^-1^). The criteria of dose escalation was based on a modified Fibonacci method with dose increased by 100, 200, 400, and 600 mg, respectively. Each patient was administered BIS orally on a fixed twice-weekly schedule (on Monday and Thursday, or Tuesday and Friday, or Wednesday and Saturday, or Thursday and Sunday, or Friday and Monday, or Saturday and Tuesday, or Sunday and Wednesday), in 4-week cycles, until progressive disease, unacceptable toxicities, or withdrawal of consent.

The study objectives were to determine the maximum tolerated dose based on incidence of dose-limiting toxicities (DLTs), PKs of single and multiple dosing, safety/tolerability, and preliminary efficacy of BIS in patients with R/R MM.

BIS tablets (50, 200 mg) were produced by Beijing Yiling Bio-engineering Technology Company Limited.

The study was organized and managed by Shanghai Clinical Research Organization for Medicines.

### Patients

Adult patients with a confirmed diagnosis of R/R MM who had progressed on/after two prior lines of therapies were enrolled. The study was designed in accordance with the International Conference on Harmonisation Harmonised Tripartite Guidelines for Good Clinical Practice. The protocol was approved by the institutional review board/independent ethics committee at Renji Hospital, and informed consent was obtained from all patients.

Key inclusion criteria were: 18–70 years old; measurable disease; good performance status (Eastern Cooperative Oncology Group 0–2); adequate bone marrow function (hemoglobin ≥ 80 g/L, platelet ≥ 50 × 10^9^/L, absolute neutrophil count ≥ 1.0 × 10^9^/L); good baseline organ function (AST, ALT, and total bilirubin ≤ 1.5 × ULN; serum creatinine ≤ 1.5 × ULN; or glomerular filtration rate ≥ 50 mL/min).

Key exclusion criteria included: nonsecretory MM or plasma cell leukemia; another concomitant or prior malignancy; ≥grade 2 peripheral neuropathy; significant cardiovascular disease; active HIV, hepatitis B or C infection; known central nervous system disease; pregnancy or breast-feeding; or other situations that investigator considers are inappropriate for this study.

### Study assessments

#### Pharmacokinetics

PKs of BIS were assessed after single and multiple doses administration of BIS. Blood samples (2.5 mL/sample) were collected into EDTA-K2 tubes pre dose and at 0.25, 0.5, 1, 1.5, 2, 3, 5, 7, 10, 24, 48, and 72 h post dose on day 1 and day 28. Additional samples for *C*_trough_ were collected pre dose on day 7, 14, and 21. After centrifugation at 1500 × *g* for 10 min at 4 °C, the plasma samples were separated and stored at approximately −20 °C until further analysis.

The concentrations of BIS in plasma were determined using a validated HPLC/MS/MS method [15]. The BIS response was linear (*r* > 0.998) over the plasma concentration range of 2.00–2000 ng/mL. The intra- and interday precision (RSD) were <5.2% and 6.2%, respectively, and the accuracy values were between −1.1% and 4.3%.

The PK parameters were calculated using NCA method with Phoenix WinNonlin 7.0 (Certara, Princeton, NJ, USA).

The maximum concentration (*C*_max_) and the time to reach *C*_max_ (*t*_max_) were directly acquired from the plasma concentration–time curves. The apparent elimination half-life (*t*_1/2_) was calculated as 0.693/*λ*_*z*_, where *λ*_*z*_ was the elimination rate constant calculated by linear regression of the terminal linear portion of the ln-concentration–time curve. The area under the plasma concentration–time curve (AUC) from time 0 to the last time point (AUC_0–*t*_) was calculated using the linear trapezoidal rule method. The AUC from 0 to infinity (AUC_0–∞_) was calculated as AUC_0–*t*_ + *C*_*t*_/*λ*_*z*_, where *C*_*t*_ is the last measurable concentration. Oral clearance (CL/*F*) was calculated as dose/AUC_0-∞_, and apparent volume of distribution (*V*_d_/*F*) was obtained by dividing CL/*F* by *λ*_*z*_.

Graphical assessment was used due to limited number of patients per cohort and high interindividual variability. Dose proportionality was confirmed by linear regression analyses of the ln-transformed AUC and *C*_max_ values as a function of dose.

#### Efficacy and safety assessment

Responses were based on investigator’s assessment according to IMWG criteria [16]. Disease responses were assessed every 28 days. Efficacy was assessed by ORR (at least partial response) and clinical benefit rate (at least minimal response).

Safety was evaluated continuously by physical examination, laboratory tests, and reports of adverse events (AEs) using National Cancer Institute Common Terminology Criteria for Adverse Events version 4.0.

#### Statistical analysis

Descriptive statistics were used to summarize and assess safety variables, including AEs, laboratory assays, vital signs, and electrocardiograms. AEs are summarized by their reported frequencies and percentages.

## Results

### BIS induces significant anti-MM activity as single agent in vitro

**Fig. 1:**
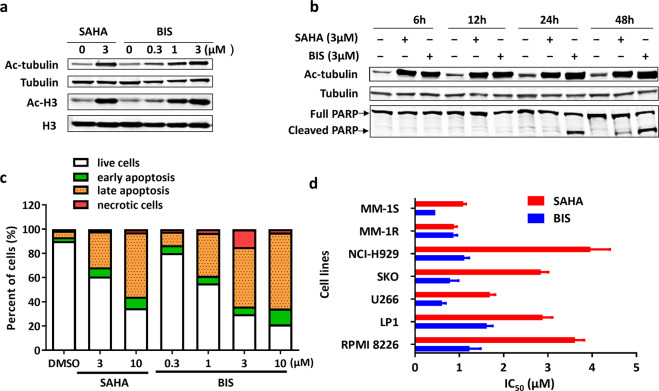
Bisthianostat (BIS) induces dose-dependent cytotoxicity in MM cells. **a** RPMI-8226 cells were cultured with control medium or BIS (0–3 μM) or SAHA (0–3 μM) for 20 h. Whole-cell lysates were subjected to Western blotting for the change of acetylated α-tubulin and H3. **b** RPMI-8226 cells were cultured with control medium or 3 μM BIS or SAHA for different time. Whole-cell lysates were subjected to Western blotting for the change of acetylated α-tubulin and PARP cleavage. **c** RPMI-8226 cells were treated with BIS (0–10 μM) or SAHA (0–10 μM) for 48 h. Cell apoptosis was evaluated with Annexin V/PI FACS assay. **d** Seven MM cell lines were treated with increasing doses of BIS or SAHA for 72 h, and cell viability was measured by CCK8 assay, and the IC_50_ was calculated with GraphPad.

BIS has been reported as a pan-HDACi with IC_50_ of 0.095–0.221 µM against HDAC1/2/3/4/5/6/8/10/11, more potent than SAHA, but without activity against HDAC7/9 (Table 1) [14]. Moreover, BIS showed great enzyme inhibition selectivity against other non-HDAC enzymes such as SIRT1/2/3 (Supplementary Table 1), with the IC_50_ over 200 μM. Here, the effect of BIS on the HDAC activity in MM cells was firstly evaluated, and the alteration of acetylation of H3 and tubulin was detected as before reported [17]. After RPMI-8226 cells were treated for 20 h, BIS could enhance the acetylation of H3 and tubulin in a dose-dependent manner, and show more potent H3 acetylation inducing ability than SAHA (Fig. 1a). Then, RPMI-8226 cells were treated with BIS or SAHA (3 μM) for different times. As Fig. 1b shown, the acetylation of tubulin increased as early as 6 h treatment, and the cell apoptosis molecular marker of PARP cleavage appeared at 24 h treatment. Accordingly, BIS also induced RPMI-8226 cell apoptosis in dose-dependent manner from 0.1 to 10 μM via FACS annexin V-FITC/PI staining assay, and showed more apoptosis induction ability than SAHA, at the same concentration. In order to test the sensitivity of MM cells to BIS, a panel of seven MM cell lines (MM.1S, MM.1R, LP1, H929, SKO, RPMI-8226, U266) was exposed to different concentrations of BIS or SAHA for 3 days. BIS triggered significant dose-dependent inhibition of cell proliferation with the 50% inhibitory concentration (IC_50_) in the range of 0.32–1.62 μM (Fig. 1c). MM.1R and MM.1S cells showed the lowest tolerance (IC_50_: 0.32, 0.46 μM). The IC_50_ of SAHA is about 2–3 times higher than BIS (0.88–4.04 μM). Collectively, BIS showed more potent HDAC and MM cell growth inhibition than SAHA.

**Table 1:** The IC_50_ of HDAC family inhibition of bisthianostat in vitro.

HDAC	IC_50_ (μM)	
	BIS	SAHA
HDAC1	0.095 ± 0.049	0.211 ± 0.114
HDAC2	0.193 ± 0.065	0.474 ± 0.142
HDAC3	0.108 ± 0.060	0.232 ± 0.147
HDAC4	0.158 ± 0.048	0.354 ± 0.126
HDAC5	0.138 ± 0.051	0.311 ± 0.092
HDAC6	0.068 ± 0.030	0.180 ± 0.086
HDAC7	>20	>20
HDAC8	0.221 ± 0.136	0.415 ± 0.233
HDAC9	>20	>20
HDAC10	0.171 ± 0.094	0.410 ± 0.242
HDAC11	0.141 ± 0.023	0.609 ± 0.580

### BIS induces significant anti-MM activity as single agent in vivo

**Fig. 2:**
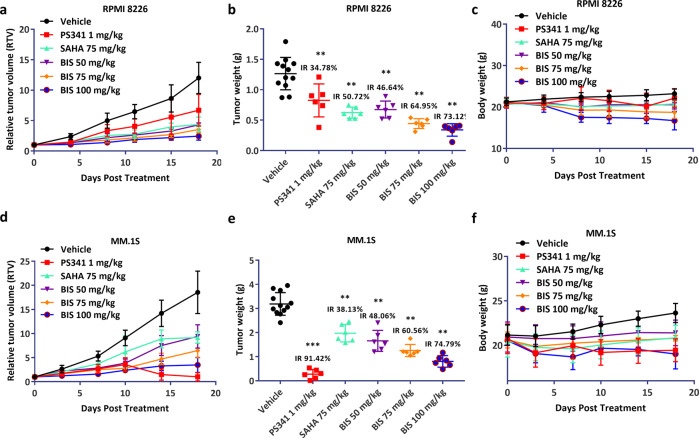
Bisthianostat induces significant anti-MM activity in vivo. **a**–**c** RPMI-8226 plasmacytoma model was treated with vehicle (*n* = 12), BIS (50, 75, 100 mg·kg^-1^·d^-1^, BID, *n* = 6), SAHA (75 mg·kg^-1^·d^-1^, BID, *n* = 6), and bortezomib (1 mg·kg^-1^·d^-1^, i.v., D1/4, *n* = 6) for 18 days. Tumor growth volume (**a**) and tumor weight (**b**) were significantly inhibited in the BIS treated group compared with controls, in dose-dependent manner, and BIS treatment did not significantly affect the body weight of the animals (**c**). **d**–**f** MM.1S plasmacytoma model was treated with vehicle (*n* = 12), BIS (50, 75, 100 mg·kg^-1^·d^-1^, BID, *n* = 6), SAHA (75 mg·kg^-1^·d^-1^, BID, *n* = 6), and bortezomib (1 mg·kg^-1^·d^-^^1^, i.v., D1/4, *n* = 6) for 18 days. Tumor growth volume (**d**) and tumor weight (**e**) were significantly inhibited in the BIS treated group compared with controls, in dose-dependent manner. BIS treatment did not significantly affect the body weight of the animals (**f**).

We next evaluated the in vivo anti-MM activity of BIS using two human MM xenograft models. NOD/SCID mice bearing s.c. RPMI-8226 or MM.1S human xenografts were treated with BIS (50, 75, 100 mg·kg^-1^·d^-1^ bid, oral, *n* = 6 mice) or vehicle alone (*n* = 12 mice) for 18 consecutive days. Significant inhibition of tumor growth (T/C (%): 35.82, 29.49, 20.28, respectively) was noted in mice bearing RPMI-8226 xenograft treated with 50, 75, 100 mg·kg^-1^·d^-1^ bid BIS compared to vehicle treated controls (Fig. 2a). Accordingly, the percent of tumor weight inhibition is 46.64%, 64.95%, and 73.12% was observed (Fig. 2b). The 75 mg·kg^-1^·d^-1^ bid SAHA treatment also induced significant tumor growth inhibition with the T/C (%) of 36.37, and tumor weight inhibition with 50.72%. In parallel, the anti-MM activity of increasing dose of BIS was evaluated in MM.1S xenografts model. Similar tumor growth inhibition was noted. The T/C (%) of 50, 75, 100 mg·kg^-1^·d^-1^ bid BIS treatment is 50.27, 34.88, and 18.75, respectively (Fig. 2d). And the percent of tumor weight inhibition is 48.06%, 60.56%, and 74.79% (Fig. 2e). The 75 mg·kg^-1^·d^-1^ bid SAHA treatment induced significant tumor growth inhibition with the T/C (%) of 49.40, and tumor weight inhibition with 38.13%. In both animal models, no significant weight loss was seen in BIS, SAHA, and PS-341 treated animals versus control (Fig. 2c, f). Taken together, these data clearly demonstrate that BIS exerts a significant in vivo anti-MM activity, better than SAHA.

**Fig. 3:**
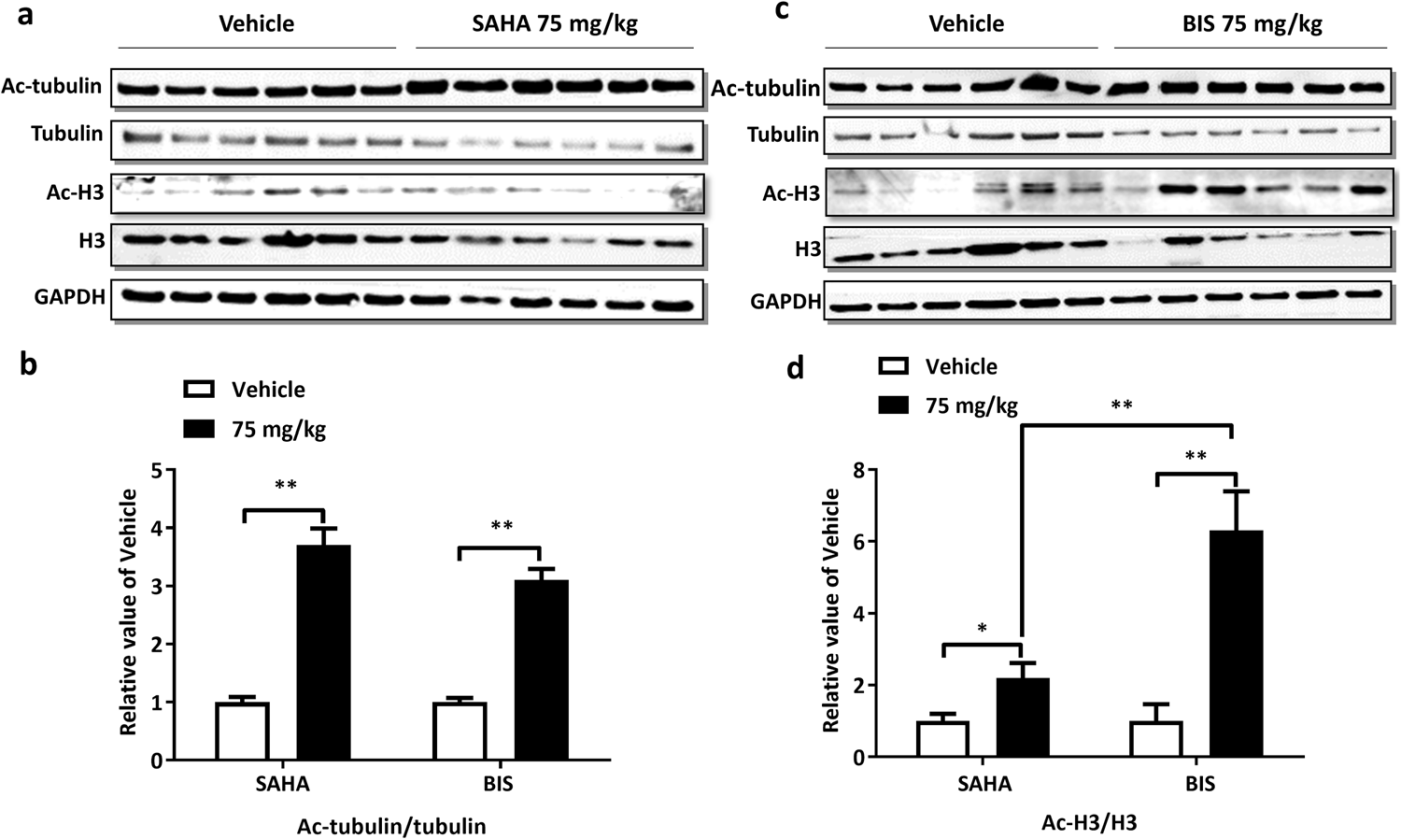
Acetylation of α-tubulin and H3 profile of bisthianostat in vivo. MM.1S plasmacytoma model was treated with vehicle (*n* = 12), BIS (75 mg·kg^-1^·d^-1^, BID, *n* = 6), and SAHA (75 mg·kg^-1^·d^-1^, BID, *n* = 6) for 18 days. Tumor was excised from each mouse at 1 h after the last dose. Western blotting analysis showed a significant increase in acetylation of α-tubulin and H3 after administration of SAHA (**a**) and BIS (**c**).  The fold change in acetylation of α-tubulin (**b**) and H3 (**d**) relative to baseline was calculated with Odyssey V3.0 (*P < 0.05, **P < 0.01).

Accordingly, the acetylation status of H3 and tubulin was evaluated at the end of MM.1S xenografts experiment. At 1 h after last dose, both of BIS and SAHA 75 mg·kg^-1^·d^-1^ bid treatment could induce significant enhancement of acetylated α-tubulin and acetylated histone H3 levels in MM.1S tumor tissues. And BIS-induced acetylated histone H3 level was higher than SAHA with significant difference (Fig. 3).

### Combining BIS with bortezomib induces significant anti-MM activity in vitro and in vivo

**Fig. 4:**
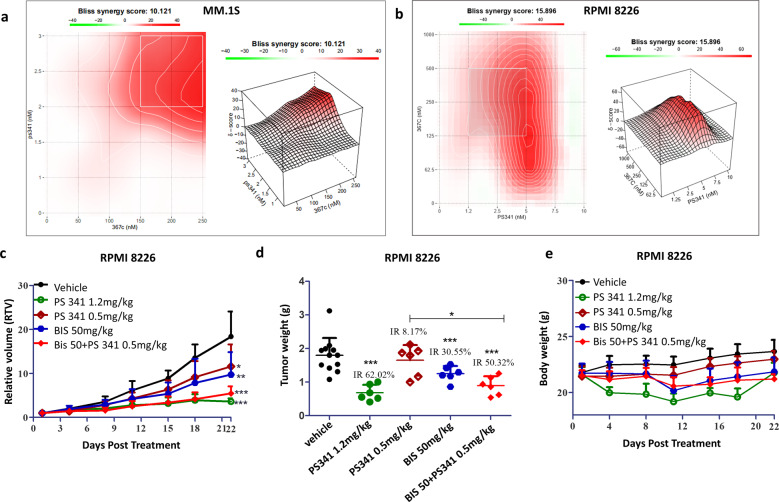
Bisthianostat in combination with bortezomib induces significant anti-MM activity in vitro and in vivo. BIS in combination with bortezomib showed clear synergistic effect in MM.1S (**a**) and RPMI-8226 cells (**b**). Cells were treated with BIS and bortezomib, alone and in combination for 72 h, cell viability was measured by CCK8 assay, and the synergistic effect was calculated, according to the BLISS method of SynergyFinder [18]. **c**–**e** RPMI-8226 plasmacytoma model was treated with vehicle (*n* = 12), BIS (50 mg·kg^-1^·d^-1^, BID, *n* = 6), and bortezomib (0.5, 1.2 mg·kg^-1^·d^-1^, i.v., D1/4, *n* = 6) or the combination of BIS plus bortezomib (0.5 mg·kg^-1^·d^-1^, *n* = 6) for 22 days. Tumor growth volume (**c**) and tumor weight (**d**) were significantly inhibited in the combination-treated group compared with BIS or bortezomib single treatment group (*P < 0.05, **P < 0.01, ***P < 0.001 vs vehicle). And combination treatment did not significantly affect the body weight of the animals (**e**).

We tested the effects of simultaneous treatment of MM cells (cell lines MM.1S and RPMI-8226) with BIS and bortezomib according to the BLISS method of SynergyFinder [18]. The combination of BIS with bortezomib showed strong synergistic effects from low to high concentration in both MM.1S and RPMI-8226 cells (Fig. 4a, b). Then, we further test the in vivo combination effect of BIS with bortezomib in RPMI-8226 xenografts model. Xenografted mice were treated with vehicle (*n* = 12), BIS (50 mg/kg, *n* = 6), bortezomib (0.5 mg/kg, *n* = 6) or the combination of BIS plus bortezomib (*n* = 6) for 22 days. Tumor growth and tumor weight were significantly inhibited in the combination-treated group compared with controls (*P* < 0.001) or compared with either agent alone (*P* < 0.05) (Fig. 4c, d). And the combination treatment did not significantly affect the body weight of mice (Fig. 4e).

### BIS PK, tissue distribution profile in mice, and CYPs inhibition assessment

Preclinical PK study (Table 2) showed BIS was quickly absorbed and cleared in mice (50, 100, and 200 mg/kg, *p.o.*) with a *t*_max_ = 0.25–0.5 and *t*_1/2_ = 1.03–1.63 h. The oral bioavailability was moderate with *F*(%) = 35.5%, 27.6%, and 16.9% for 50, 100, and 200 mg/kg, respectively. The CL in mice was 1.37 L·h^-1^·kg^-1^ following 10 mg/kg i.v. dose.

**Table 2:** The pharmacokinetic profile of bisthianostat in mice.

Dose	Gender	*t*_max_	*C*_max_	S.E. for *C*_max_	AUC_0–*t*_	S.E. for AUC_0–*t*_	AUC_0–∞_	MRT_0–∞_	*t*_1/2_	CL	*V*_ss_
(mg/kg)		(h)	(μg/mL)	(μg/mL)	(h × μg/mL)	(h × μg/mL)	(h × μg/mL)	(h)	(h)	(L·h^-1^·kg^-1^)	(L/kg)
50	Male	0.25	8.34	0.48	12.1	0.68	12.8	1.35	0.96	/	/
(*p.o.*)	Female	0.25	7.97	0.51	14.1	0.60	14.1	1.86	1.10	/	/
	Mean^a^	0.25 (0.25–0.25)	8.15 (3.13%)	/	13.0 (10.9%)	/	13.4 (7.1%)	1.61 (22.4%)	1.03 (9.6%)	/	/
100	Male	0.50	8.68	1.54	15.8	1.18	16.0	1.96	0.77	/	/
(*p.o.*)	Female	0.50	8.65	1.12	25.9	2.12	26.0	3.45	1.13	/	/
	Mean^a^	0.50 (0.50–0.50)	8.66 (0.24%)	/	20.2 (35.9%)	/	20.4 (35.5%)	2.71 (38.8%)	0.95 (26.6%)	/	/
200	Male	0.50	8.88	0.68	23.3	2.28	23.8	2.52	1.82	/	/
(*p.o.*)	Female	0.25	10.8	2.05	26.4	2.37	26.5	2.85	1.46	/	/
	Mean^a^	0.38 (0.25–0.50)	9.80 (14.1%)	/	24.8 (8.9%)	/	25.1 (7.7%)	2.68 (8.1%)	1.63 (15.5%)	/	/
10	Male	/	/	/	6.80	0.18	6.81	0.22	0.26	1.47	0.33
(i.v.)	Female	/	/	/	7.88	0.39	7.90	0.24	0.29	1.27	0.30
	Mean^a^	/	/	/	7.32 (10.5%)	/	7.34 (10.6%)	0.23 (3.4%)	0.27 (7.1%)	1.37 (10.5%)	0.31 (7.1%)

The parameters for male and female mice are calculated based on the mean concentration–time profiles and the standard error (SE) for *C*_max_ and AUC_0–*t*_ is provided additionally. / denotes not available.

^a^The *t*_*max*_ is presented by median (range), *C*_max_ and AUC are presented by geometric mean (CV%), and others are presented by mean (CV%).

**Fig. 5:**
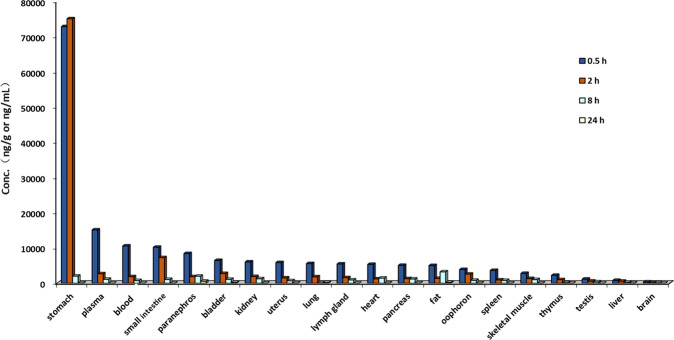
The tissue distribution of bisthianostat in mice. Tissues were collected and analyzed after 100 mg/kg (*p.o.*) treatment at 0.5, 2, 8, and 24 h.

Except stomach and intestine, tissue distribution results showed that BIS was favorably distributed in blood compared with other tissues such as lung and liver (Fig. 5) as indicated by its *V*_ss_ value as 0.31 L/kg. This distribution parameter might contribute to its in vivo efficacy to treat hematologic neoplasms such as MM.

Major cytochrome P450s inhibition assay was carried out using specific probe substrates in the presence of human liver microsomes and the results are summarized in Supplementary Table 2. Analysis data showed that BIS displayed moderate inhibition (IC_50_ = 4.76 μM) toward CYP1A2 and weak inhibition (IC_50_ = 32.4 μM, midazolam 1′-hydroxylation; IC_50_ = 20.8 μM, testosterone 6β-hydroxylation) for CYP3A4. No inhibition (IC_50_ > 100 μM) was observed toward other human CYP isoforms including CYP2B6, CYP2C8, CYP2C9, CYP2C19, and CYP2D6. Its main metabolite M351 showed no inhibition toward major cytochrome P450s (IC_50_ > 100 μM).

### PK profile of BIS in MM patients

**Fig. 6:**
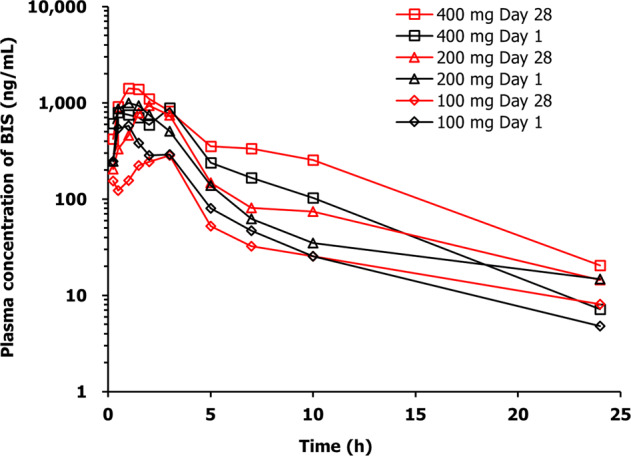
The geometric mean concentration-time curve of bisthianostat in MM patients following single and multiple doses oral administration.

The PK profile and mean plasma concentration–time curve of BIS in MM patients following single and multiple doses administration are shown in Table 3 and Fig. 6.

**Table 3:** The pharmacokinetic profile of bisthianostat in MM patients.

	Group	*n*	*t*_max_	*C*_max_	AUC_0–*t*_	AUC_0–∞_	*t*_1/2_	CL/*F*	*V*_d_/*F*
			(h)	(μg/mL)	(h × μg/mL)	(h × μg/mL)	(h)	(L/h)	(L)
Day 1	100 mg	3	0.5 (0.5–1.0)	0.599 (61.7%)	2.04 (45.2%)	2.07 (45.4%)	4.28 (31.2%)	51.6 (46.4%)	314 (45.3%)
	200 mg	3	0.5 (0.5–1.5)	1.11 (29.4%)	3.63 (22.6%)	3.76 (20.0%)	4.38 (15.0%)	53.8 (18.6%)	343 (28.2%)
	400 mg	2	2.25 (1.25-3.0)	1.30 (14.8%)	5.13 (12.6%)	5.19 (13.8%)	4.10 (41.3%)	77.5 (13.7%)	445 (28.4%)
Day 28	100 mg	3	1.5 (1.5–3.0)	0.544 (26.8%)	1.67 (40.8%)	1.77 (44.1%)	6.30 (44.2%)	59.8 (36.2%)	487 (18.1%)
	200 mg	3	1.0 (1.0–3.0)	1.32 (61.5%)	4.48 (33.2%)	4.76 (27.5%)	7.99 (55.4%)	43.0 (25.0%)	538 (72.8%)
	400 mg	2	1.0 (1.0–1.0)	1.41 (26.4%)	9.42 (40.6%)	9.63 (37.5%)	3.47 (39.2%)	42.9 (35.5%)	230 (69.9%)

The parameters *C*_max_ and AUC are presented by geometric mean (CV%), *t*_max_ is presented by median (range), and others are presented by mean (CV%).

BIS was rapidly absorbed following oral administration, with the *C*_max_ reaching at 0.5–2.25 h. Then, it was eliminated quickly from plasma, with a half-life of about 4 h. After a single administration, the geometric mean of *C*_max_ was 0.599, 1.11, and 1.30 μg/mL at dose levels of 100, 200, and 400 mg, respectively, and that of AUC_0−t_ was 2.04, 3.63, and 5.13 h × μg/mL, respectively. The AUC_0−t_ and *C*_max_ exhibited dose proportionality over the range of 100–200 mg, but they increased slightly lower than dose-proportional manner from 200 to 400 mg. The mean Cl/*F* was from 51.6 to 77.5 L/h, and *V*_d_/F was from 314 to 445 L/h. Similar results were observed after multiple doses administration. BIS was not accumulated following multiple doses administration, as indicated by the accumulation index similar to 1. The values of *C*_trough_ on days 7, 14, and 21 were all below the lower limit of quantification set at 2.00 ng/mL. Based on these findings and the short elimination half-life of BIS, it is assumed that  BIS could achieve the steady state within 7 days.

### The preliminary results of ongoing clinical study

As of the data cut-off date, eight patients were enrolled at three dose levels from 100 to 400 mg of BIS. The median age at enrollment was 62 years (range 51–70 years). The median number of previous lines of therapy was 5 (range 2–6). Per protocol, all of eight patients were evaluable for toxicities, PK, and efficacy.

Any grade hematological treatment-related AEs occurred in four of eight patients (50%), while grade 3/4 hematological AEs occurred in two (25%) patients. Any grade nonhematological treatment-emergent AEs were observed in three (37.5%) patients; no grade 3/4 nonhematological AEs were reported. No patient discontinued the treatment of BIS due to AEs. Except one patient in 200 mg cohort experienced a grade 2 nausea and vomiting, no other patients experienced diarrhea, nausea, or vomiting. Overall single-agent efficacy was modest, SD was observed in four (50%) patients as best response at the end of first dosing cycle (day 28), one of which remained stable for up to nearly 7 months before progression of myeloma. At the time of data cut-off for statistical analysis, no DLT has been observed. At present, this phase 1a clinical trial is still in progress. Further studies will be conducted to assess the safety and efficacy in a more frequency schedule based on current data.

## Discussion

HDAC is typically comprised of a surface recognition cap moiety that can tolerate extraordinary variability, a linker group that traverses the tunnel of the active site and a zinc-binding group (ZBG) that chelates active site zinc ion (Zn^2+^) [19]. BIS contains hydroxamic acid as the ZBG, which is the most commonly used and succeeded ZBG in the structure of HDACis, such as FDA-approved drug vorinostat, panobinostat, and belinostat.

To investigate the inhibitory effect of BIS on HDACs activity, we evaluated its effect on the acetylation of α-tubulin, the HDAC6’s substrate, and the acetylation of lysine on histone H3, the other HDAC’s substrate. BIS induces potent acetylation of both α-tubulin and H3 as low as 0.32 μM, confirming its potent inhibitory effect on pan-HDACs activity, in dose and time dependent manner, better than SAHA. Correspondingly, the apoptosis marker, the PARP cleavage appeared at 24 and 48 h after treatment (Fig. 1b), and cell apoptosis showed a dose-dependent manner, after BIS treatment for 48 h, better than SAHA (Fig. 1c). Then, BIS showed dose-dependent inhibition of cell proliferation in all of seven MM cell lines, with the IC_50_ of 0.32–1.62 μM, about 2–3 times lower than SAHA (0.88–4.04 μM) (Fig. 1d). BIS showed more potent anti-HDACs activity at molecular and cellular level for MM cell growth inhibition and cell apoptosis than SAHA. We also evaluated its in vivo anti-MM effect in two different plasmacytoma xenograft models. BIS triggered significant anti-MM activity in dose-dependent manner, clearly demonstrating the preclinical potent efficacy of candidate drug BIS as a single agent in MM disease. Next, the combination effect of BIS with bortezomib was evaluated. A significant decrease in MM cell viability was observed with combined treatment, compared with single agent alone, demonstrating potent synergism in consistence with previous report on panobinostat [20]. Furthermore, the combination effect was further confirmed in the xenograft mice models.

BIS was chosen as a drug candidate because of its properties that favor drug development, not only including good potent activity against HDAC family and strong selectivity against other non-HDAC enzyme, such as SIRTs and kinases (Table 1 and Supplementary Table 1), but also good oral bioavailability and PK properties in rodents and nonrodents, minimal potential for QTc prolongation (IC_50_ > 40 μM for hERG), and well tolerance in animal studies.

HDACis with hydroxamic acid as ZBG have poor PKs such as rapid degradation and clearance in vivo [21]. Vorinostat possessed high serum clearance, a short elimination half-life, and low oral bioavailability in rat and dog [22]. Belinostat has rapid clearance with a *t*_1/2_ of 1.6 h [23]. Similar to other hydroxamic acid HDACis, the oral PK of BIS also showed rapid absorption and rapid clearance with the *t*_max_ of 0.25–0.5 h and *t*_1/2_ of 1.03–1.63 h, but higher *C*_max_ and AUC.

Except the PK property, drug distribution profile also plays a key role in determining pharmacologic response [24]. BIS was favorably distributed in blood compared with other tissues such as lung and liver, except stomach and intestine. In contrast, the highest exposure of panobinostat (by *C*_max_ or AUC) was in the bile, stomach, intestine, and liver. The distribution of panobinostat in blood was the lowest, except muscle and fat. This preferred blood distribution parameter of BIS might contribute to its in vivo efficacy to treat hematologic neoplasms with relative low incidence of nonhematological toxicity.

Cardiac toxicity is one of the major side effects/concerns preventing progress of HDACi in the clinics [19]. Panobinostat carries a boxed warning for severe and fatal cardiac ischemic events, severe arrhythmias, and ECG changes [25]. The most accepted mechanism for the HDACi-induced QT interval prolongation is the inhibition of hERG. Panobinostat inhibited hERG with IC_50_ values of 3.1 μM [26]. Efforts were explored to improve the selectivity of HDACi with weak affinity for the hERG [27]. The IC_50_ value of BIS in the hERG assay was up to over 40 μM, which would lower the potential possibility of QT prolongation.

However, hERG channel inhibition is just one potential mechanism of QT prolongation, and QT prolongation is not a straightforward correlate of ventricular fibrillation. SB939, another hydroxamate-based HDACi, showed evidence of QT prolongation during phase one clinical trials, but did not bind to hERG channels up to 10 μM [28, 29]. Although SAHA did not affect hERG K^+^ channels up to 300 μM [26], but it showed conflicting evidence of QT prolongation during clinical trials [30]. So, the effect of HDACi on the induction of QT prolongation is needed to be further elucidated in clinical trial.

The first-in-human PK data of BIS (Table 3) show good absorption with *t*_max_ of 0.5–2.25 h, and high exposures with AUC over 2 h × μg/mL in relative linear PKs from 100 to 200 mg. It is eliminated rapidly with a half-life of about 4 h. Whereas panobinostat has longer *t*_1/2_ of 16 h, but lower exposure [31]. One patient at 400 mg cohort showed a high exposure on day 28, which was caused by a multiple-peak PK profile. It is unclear why this patient has such a different profile from others. As there is a relatively large variability in MM patients, more data are needed to precisely depict the PKs profiles of BIS.

The preliminary results of first-in-human findings from CH-020PI study, an ongoing phase 1a study, showed that BIS was well absorbed and tolerated in patients with R/R MM. As of the data cut-off date, no grade 3/4 nonhematological AEs were reported. No patient discontinued the treatment due to AEs. Except one patient in 400 mg cohort experienced a grade 2 nausea and vomiting 3 days after second oral dose, no other patients experienced diarrhea, nausea, or vomiting. It is worthy to be noted that gastrointestinal toxicity is common with the use of HDACi, including panobinostat [32]. In addition, it is encouraging that no clinical cardiac toxicity has been observed in our cohort. In the previously reported data, the incidence of cardiovascular toxicity is relatively frequent in cancer patients receiving other HDACi treatment [33]. Although modest activity was observed, other studies of single-agent deactylase inhibitors in R/R MM including panobinostat, vorinostat, and romidepsin have yielded similar results [34–36].

In conclusion, the results from our in vitro and in vivo preclinical studies showed a significant anti-MM activity of BIS and a synergistic effect in combination with bortezomib. The preliminary first-in-human study showed single-agent BIS was a promising HDACi drug with a good safety window for patients with R/R MM. This phase 1a study is still in progress to further identify the optimal dose/schedule of BIS monotherapy and clinical investigation of BIS in combination with other anti-myeloma agents, including proteasome inhibitors and Dex in patients with R/R MM is therefore worth developing.

## Supplementary information

Supplementary Table 1

Supplementary Table 2
