# Supplementary material for: Pharmacodynamic, pharmacokinetic, and phase 1a study of bisthianostat, a novel histone deacetylase inhibitor, for the treatment of relapsed or refractory multiple myeloma
Source: Acta Pharmacol Sin. 2021 Aug 2;43(4):1091–9. doi: 10.1038/s41401-021-00728-y (PMC8976035; doi:10.1038/s41401-021-00728-y)
Supplement: Supplementary file 1 — Supplementary Table 1 [file 41401_2021_728_MOESM1_ESM.docx]

**Supplemental Table 1**

| Model | Inhibition rate (%) (200 μM) | |
| --- | --- | --- |
|  | BIS | SAHA |
| SIRT1 | 38.9±1.7 | 7.6±8.8 |
| SIRT2 | 44.1±1.1 | 43.6±0.8 |
| SIRT3 | 38.7±3.1 | 27.8±9.2 |
| MMP12 | 45.3±1.5 | 61.1±3.3 |
| DPP4 | 48.5±4.5 | 11.3±5.4 |
| Caspase3 | 41.3±3.6 | 3.9±2.5 |
| PKCepsilon | 10.7±8.1 | 0.2±5.7 |
| AuroraA | 20.8±3.4 | 18.8±7.8 |
| Braf | 22.3±10.6 | 17.6±9.8 |
| Syk | -0.7±4.1 | 13.7±1.8 |
| FBP | 54.0±4.4 | -4.1±0.6 |
| PTP1B | 10.1±6.6 | 9.3±6.2 |
