# Supplementary material for: Pharmacodynamic, pharmacokinetic, and phase 1a study of bisthianostat, a novel histone deacetylase inhibitor, for the treatment of relapsed or refractory multiple myeloma
Source: Acta Pharmacol Sin. 2021 Aug 2;43(4):1091–9. doi: 10.1038/s41401-021-00728-y (PMC8976035; doi:10.1038/s41401-021-00728-y)
Supplement: Supplementary file 2 — Supplementary Table 2 [file 41401_2021_728_MOESM2_ESM.docx]

**Supplemental Table 2**

| CYP isoform | Bisthianostat  concentration (μM) | Substrate  concentration (μM)^a^ | Remaining enzyme activity (%) | CYP isoform | Bisthianostat concentration (μM) | Substrate concentration (μM) ^a^ | Remaining enzyme activity (%) |
| --- | --- | --- | --- | --- | --- | --- | --- |
| CYP1A2 | 0 | 2.42 | 100 | CYP2C9 | 0 | 0.96 | 100 |
|  | 0.10 | 2.42 | 100 |  | 0.10 | 1.00 | 104 |
|  | 0.33 | 2.28 | 94.4 |  | 0.33 | 0.87 | 90.4 |
|  | 1.00 | 1.93 | 80.0 |  | 1.00 | 0.94 | 98.4 |
|  | 3.33 | 1.37 | 56.6 |  | 3.33 | 0.93 | 96.9 |
|  | 10.0 | 0.70 | 28.9 |  | 10.0 | 0.86 | 89.6 |
|  | 33.0 | 0.33 | 13.6 |  | 33.0 | 0.79 | 82.1 |
|  | 50 | 0.18 | 7.52 |  | 50 | 0.74 | 77.5 |
| CYP2D6 | 0 | 0.45 | 100 | CYP2C8 | 0 | 0.24 | 100 |
|  | 0.10 | 0.41 | 90.0 |  | 0.10 | 0.23 | 97.1 |
|  | 0.33 | 0.40 | 88.1 |  | 0.33 | 0.22 | 95.4 |
|  | 1.00 | 0.39 | 87.4 |  | 1.00 | 0.25 | 105 |
|  | 3.33 | 0.41 | 91.3 |  | 3.33 | 0.23 | 97.2 |
|  | 10.0 | 0.41 | 92.0 |  | 10.0 | 0.23 | 99.5 |
|  | 33.0 | 0.40 | 87.8 |  | 33.0 | 0.20 | 83.7 |
|  | 50 | 0.43 | 96.3 |  | 50 | 0.13 | 55.9 |
| CYP2C19 | 0 | 0.26 | 100 | CYP2B6 | 0 | 0.29 | 100 |
|  | 0.10 | 0.25 | 96.2 |  | 0.10 | 0.28 | 95.4 |
|  | 0.33 | 0.24 | 92.6 |  | 0.33 | 0.26 | 91.3 |
|  | 1.00 | 0.24 | 93.4 |  | 1.00 | 0.26 | 90.3 |
|  | 3.33 | 0.24 | 93.4 |  | 3.33 | 0.26 | 90.3 |
|  | 10.0 | 0.23 | 88.4 |  | 10.0 | 0.26 | 90.2 |
|  | 33.0 | 0.20 | 78.6 |  | 33.0 | 0.27 | 92.9 |
|  | 50 | 0.15 | 60.3 |  | 50 | 0.27 | 94.6 |
| CYP3A4^b^ | 0 | 0.74 | 100 | CYP3A4c | 0 | 79.5 | 100 |
|  | 0.10 | 0.59 | 79.4 |  | 0.10 | 78.3 | 98.5 |
|  | 0.33 | 0.68 | 91.3 |  | 0.33 | 73.6 | 92.6 |
|  | 1.00 | 0.58 | 77.8 |  | 1.00 | 70.5 | 88.7 |
|  | 3.33 | 0.60 | 81.6 |  | 3.33 | 67.9 | 85.4 |
|  | 10.0 | 0.52 | 69.7 |  | 10.0 | 56.3 | 70.8 |
|  | 33.0 | 0.33 | 44.7 |  | 33.0 | 33.1 | 41.6 |
|  | 50 | 0.23 | 30.8 |  | 50 | 14.3 | 17.9 |

^a^ Substrates are phenacetin (CYP1A2), tolbutamide (CYP2C9), dextromethorphan (CYP2D6), taxol (CYP2C8), midazolam (CYP3A4), testosterone (CYP3A4), [S-mephenytoin](https://www.sigmaaldrich.com/catalog/product/sigma/uc126) (CYP2C19) and bupropione (CYP2B6). ^b^ Substrate is midazolam. ^c^ Substrate is testosterone.
